# Exploring the quantity and type of evidence collected during criminal investigations in South Korea

**DOI:** 10.1016/j.fsisyn.2024.100544

**Published:** 2024-07-30

**Authors:** Minhwan Jang

**Affiliations:** Department of Policing and Security, Rabdan Academy, 65, Al Inshirah Al Sa'adah, Abu Dhabi, 22401, United Arab Emirates

**Keywords:** Evidence collection, Police investigation, Physical evidence, Testimonial evidence, Interrogation

## Abstract

This article explores the patterns of 11 types of criminal evidence collected by a detective team in real-life 172 volume crime cases that occurred in South Korea. Electronic and paper case files were analyzed to examine the patterns of how much and which type of evidence was collected per case. The results show that, in general, the team collected almost four pieces of evidence per case, and notably, testimonial evidence (e.g., statements) was collected more often than physical evidence (e.g., video recordings, DNA, and fingerprints). Among the physical evidence types, video recording was the most available; victim statement was the most available of testimonial evidence types. Another finding was that more evidence was collected before than after interrogation. Overall, this study provides empirical insights into the current landscape of evidence collection practices in South Korean police investigations, with implications for both law enforcement procedures and academic research on criminal justice systems.

## 1 Introduction

In police investigations, detectives can collect physical and testimonial types of criminal evidence, which is the crux of all police work. Collected evidence can influence detectives' perception of evidentiary value and subsequent decisions about whether to arrest a person, seize personal belongings, file a police charge,^1^ or use specific interviewing tactics during the interrogation. Therefore, the police's ability to secure sufficient evidence is one of the most crucial elements of the criminal justice system. However, there is a paucity of research in the literature on the trend in the type of evidence that can be collected in an investigation. Thus, in this study, I aim to explore quantitative patterns of evidence collection by analyzing 172 volume crime cases in South Korea, helping practitioners and researchers gain scientific insights and have a clearer grasp for future research and practical applications.

### 1.1 Volume crime investigation in South Korea

South Korea has a centralized policing system managed by the Korean National Police Agency (KNPA). The KNPA oversees 16 local provincial police agencies and 259 police stations strategically placed based on population size and security needs [1]. Each police station houses a criminal investigation department, typically divided into two units: a) felony and b) volume crime investigation teams. Each of these teams comprises five detectives, including four field detectives and one team leader. This organizational structure ensures that volume crimes are investigated separately from felony crimes, allowing for more focused and specialized attention in each case. Regarding crime scene investigations and collecting forensic evidence, the detectives in the criminal investigation department can decide whether crime scene investigators should be dispatched to scenes depending on the severity of the crime.

Volume crime has been broadly defined by researchers and organizations. Its definition often includes (a) crime categories that constitute a large proportion of recorded crimes (e.g., burglary, robbery, theft, fraud, criminal damage, and minor interpersonal violence; [2]), (b) clearance rates [3], or (c) the sheer volume of occurrences [4].

In the South Korean police context, the definition of volume crime is more comprehensive, incorporating all three factors mentioned above, with an additional factor: awareness of suspect's identity. Typically, criminal cases in which the suspect's identity is known to victims, patrol officers, bystanders, or reporters are assigned to the volume crime investigation teams when the crime is reported. However, exceptions exist, such as serious criminal cases with identified suspects, which are assigned to felony crime teams. It is important to note that cybercrimes occurring in online spaces (e.g., social networking platforms, Internet webpages, SMS, or phone calls) are not included in this definition.

In summary, volume crimes in South Korea encompass a considerable proportion of offline crimes, in which suspects are often identified prior to case assignment. These include personal violence, theft, fraud, property damage, unidentified decedent cases (i.e., natural and accidental death cases, and suicide), and other minor crimes.

### 1.2 Importance of physical evidence

Research has consistently shown that physical evidence, such as DNA and fingerprints, is highly valued in criminal investigations [[5], [6], [7], [58]]. Physical evidence significantly increases the likelihood of identification, arrest, case resolution, or conviction, particularly in cases of violence, burglary, robbery, and homicide [6,[8], [9], [10], [11]]. Police officers generally perceive physical evidence to be more significant than testimonial evidence [12], which applies to other groups of people such as prosecutors [[13], [14], [55]], lawyers, judges, and even laypeople [6,[15], [16], [17], [18]].

Wüllenweber and Giles [7] also reported that in volume crime investigations, the presence of forensic evidence, such as DNA, footwear, and other multiple forensic evidence, is quite effective. However, physical evidence significantly influences legal decisions and investigation outcomes by providing reliable identification of suspects and supporting prosecutions; however, its effectiveness depends on proper crime scene management, timely analysis, and integration into the investigative process [8,19]. Studies have also shown that forensic evidence, such as DNA and fingerprints, can increase the number of suspects identified, arrested, and prosecuted compared to traditional investigation methods, such as eyewitness testimonies and suspect interrogations, thereby enhancing the efficiency and effectiveness of criminal investigations [8,19]. In contrast, Peterson and colleagues [11] and Baskin and Sommers [20,21] reported that forensic evidence is not occasionally collected during police investigations, and its impact is not determinative for most crimes; therefore, physical evidence plays an auxiliary role in criminal proceedings.

### 1.3 The rising dominance of closed-circuit television (CCTV)

There has been a debate over the ethical issues of widespread surveillance and the effectiveness of CCTV technology in increasing security and deterring crime [22]; however, there is no doubt that CCTV footage has become a useful resource in police investigations, particularly for identifying and charging suspects in homicide cases [23]. In addition, CCTV footage has increased the clearance rates for crimes, particularly theft and property damage [24,25]. CCTV footage was found to be particularly beneficial in solving assault crimes that occur late at night [25]. The impact of CCTV footage can vary by offense type, such as property damage, theft, and burglary. Its admissibility in court and the challenges associated with its use, such as image quality and coverage issues, must be addressed [24].

Nonetheless, the increase in CCTV installations in South Korea can be explained by their crucial roles in crime prevention, deterrence, and clearance. The police and South Korean government are growing dependent on CCTV. Statistics on the installation and operation of CCTV by public institutions in the country [59] indicated that the number of CCTV cameras increased from 157,197 in 2008 to 1,607,388 in 2022, representing an increase of over 1000 % over the past 14 years. Furthermore, approximately 7.3 million private CCTVs have been installed [26].

KNPA [27] reported that the number of digital evidence items analyzed by the police, including personal computers, laptops, CCTV, car navigation devices, car dash cameras, smartphones, and computer files, has increased substantially from 10,426 in 2012 to 70,929 in 2022. However, the number of fingerprint examinations has stagnated from 20,486 in 2011 to 19,064 in 2022.^2^ Notably, the recovery of footprint evidence has sharply decreased from 23,541 in 2011 to 1954 in 2022. According to statistics reported by the Korean National Forensic Services [28], there has been a substantial increase in the amount of evidence analyzed, rising from 224,589 items in 2007 to 744,420 items in 2023. This encompasses a range of forensic analyses, including autopsies, chemical and biological examinations, among other forensic investigations. Notably, the analysis of DNA evidence has seen a considerable increase, with the number of analyzed DNA evidence items increasing from 52,309 in 2007 to 253,120 in 2023.

In summary, it appears that the collection of physical evidence by the South Korean police has increased overall, but certain types of evidence, such as fingerprints and footprints, have not shown growth in collection in the last decade.

### 1.4 Impact of testimonial evidence

Despite the importance of physical evidence, the influence of eyewitness testimonies on case outcomes should not be overlooked. Many studies have shown that despite the perceived power of forensic evidence, testimonial evidence is one of the most available evidence types in police investigations and is known to influence police decisions on case processing in various types of crimes [20,21,29].

Eyewitnesses are considered indispensable to investigations by police officers; however, their limited capacity to provide precise and comprehensive information is acknowledged [30]. The psychological literature has demonstrated that eyewitness testimony can be unreliable, with various factors influencing its veracity [31]. Additionally, witnesses' identification decisions in a photo lineup can influence investigators’ decisions, irrespective of their accuracy [32]. Although eyewitness testimony is a common form of evidence in court proceedings and its reliability is frequently challenged, it is essential for investigators to recognize its potential limitations and biases [31].

### 1.5 Present study

It is essential to acknowledge the exploratory nature of this study as it underscores the preliminary nature of the findings and highlights the need for future research to validate and build upon these initial explorations. This study originated from the identification of a potential gap in the research concerning the quantitative aspects of evidence gathered during police investigations. Therefore, the primary aim of this study was to investigate and analyze evidence collection patterns within police investigations, an area that has not been extensively explored. By adopting an exploratory approach, this study seeks to uncover new insights, identify emerging patterns, and offer fresh perspectives on evidence collection processes and patterns. The objectives are to lay a foundation for future hypotheses, guide subsequent research efforts, and contribute to the broader body of knowledge in the field of forensic science and criminal investigation.

## 2 Methods

### 2.1 Data sampling

The author worked as a detective in a volume crime investigation team at Yongsan police station of the Seoul Metropolitan Police Agency in South Korea. Seoul, the capital city of South Korea, has a population of 9,635,445, and Yongsan Police Station has jurisdiction over Yongsan District, a densely populated area in the center of Seoul with approximately 209,849 residents [33]. The district has 13 subway stations and one of the biggest train stations in the country.

The team comprised five detectives (four field detectives and one team leader). Initially, approximately 800 cases were assigned to the team between September 2020 and January 2022, and the team leader assigned the cases to each team member sequentially. Finally, 227 cases were assigned to the author as a main investigator through a rotation system used by the police department. This system operates by sequentially assigning each new case to the next available detective, regardless of the crime type. This ensured an equitable distribution of cases among detectives and prevented selection bias. Consequently, the assignment of cases can be considered random within the operational context of police investigation shifts. However, 57 were excluded from the final data analysis because they did not meet the minimum legal requirement for initiating an investigation and were closed without further investigation. A total of 172 cases were included in the final data analysis.

### 2.2 Variables and measures

The South Korean criminal justice system operates the Korea Information System of Criminal Justice System (KICS), where all criminal cases are reported online. Police investigators must store all investigation-related information and relevant documents in the database. The KICS then allows prosecutors and judges to electronically access case documents for prosecution and trial processes. However, the police must keep all case information on paper and send paper documents to the prosecution's office simultaneously as soon as the electronic case information is transferred. Therefore, both electronic and paper documents were used for the data collection in this study.

Examining these electronic and paper documents, I coded 77 variables in the 172 case files; however, for this study, only 15 variables were used for analyses, such as evidence type, number of collected evidence, crime type, and investigative decision (see Table 1 for more details). The data were collected after excluding any identifiable personal information (e.g., names, ID numbers, residential addresses, or other sensitive information about suspects, victims, and eyewitnesses). The initial data coding was conducted in August 2021 and ended in February 2022. I preregistered the data sampling, variable definitions, and coding information on the Open Science Framework (OSF) at https://doi.org/10.17605/OSF.IO/UB5TS. The data that support the findings of this study are openly available in [OSF] at https://doi.org/10.17605/OSF.IO/SQGZB, reference number [Master DATA (240424).xlsx].

**Table 1:** Key study variables.

Variables	Measures
Video Recording^a^	Total number of documented evidence
Voice Recording^a^	
Photo^a^	
Document^a^	
Digital Trace^a^	
DNA^a^	
Fingerprint^a^	
Chemicals^a^	
Witness Testimony^a^	
Victim Statement^a^	
Suspect Statement^a^	
Suspect's Exonerating Evidence^a^	
Category of Evidence Type^b^	0 = Physical, 1 = Testimonial
Phase of Collection^b^	0 = Before-Interrogation, 1 = After-Interrogation
Crime Type^b^	Type of crime committed
Crime Scene Processing^b^	0 = Not processed, 1 = Processed
Investigative Decision^b^	0 = Referred for prosecution^c^ (i.e., summary trial, referred for prosecution), 1 = Not referred^d^ (i.e., cold case, not referred for prosecution, not charged)

a Represents a continuous variable.

b Represents a categorical variable.

c Referred for prosecution is song-chi in Korean.

d Not referred for prosecution is bul-song-chi in Korean.

### 2.3 Procedure

#### 2.3.1 Coding evidence type

After examining the case files, I categorized all evidence pieces into 11 types: video recording, voice recording, photo, DNA, fingerprint, document, chemical, digital trace, witness testimony, suspect statement, and victim statement. Witness, suspect, and victim statements were further categorized as ‘testimonial,’ whereas the other types were categorized as ‘physical.’ These evidence types were collected in many different ways; for instance, in some cases, patrol officers or victims could submit evidence directly to the detective team; in most cases, the team could go to the crime scene and collect evidence; occasionally, suspects shared exonerating evidence with the team either before or after an interrogation.

Certain types of evidence were obtained in the form of printed documents. For example, some victims, witnesses, or even suspects submitted printed copies of CCTV footage showing the crime, transcripts of verbal interactions, bank transfer certificates, screenshots of social media chat records (such as WhatsApp, Facebook, Instagram), or evidence of the financial relationship between the suspect and victim, as they did not possess the original electronic files of the evidence. In these cases, the evidence was labelled as document, rather than being categorized as voice recordings, photographs, video recordings, or digital traces. Once the individuals submitted the original files of their evidence (e.g., video files, photo files, audio recordings, downloaded chat log files), it was classified accordingly. When the detectives collected document papers and sent them for fingerprint or DNA analysis, it was recorded as fingerprint or DNA evidence.

#### 2.3.2 Defining and Measuring evidence amount

Police investigators gather all evidence, clues, and other types of information that can be used to prove crime elements and are eventually submitted to prosecutors and judges. In this process, most information collected by the police is documented and shared in the form of a report, and the trier of fact can make decisions based on the evidence documented in investigation reports. In practice, if a piece of evidence – irrespective of being physical or testimonial – has a certain level of evidentiary value, it should be documented in a report (e.g., *Evidence #1*). Suppose that the team collected additional evidence of the same type but had a different evidentiary value, and it should be filed on a separate report (e.g., *Evidence #2*); these are typically considered independent evidence of each other in legal systems.

Applying the same logic to this study, I counted each type of evidence as one piece, as long as the evidence was documented separately. For testimonial evidence, the same logic applied: When there were two different eyewitnesses, although they provided the same information to the police, they were counted separately, as their statements were recorded in different documents; these eyewitness statements were coded 2. When evidence was absent, it was coded 0; for instance, when no chemical evidence was found, it was coded 0. Additionally, none of the collected evidence was counted multiple times, meaning that each evidence was coded under a single evidence type. No forensic evidence such as fingerprint, DNA, and chemical was processed using multiple methods of analysis (i.e., a piece of fingerprint evidence was not again analyzed for further DNA testing).

Although some may argue that quantifying eyewitness statements, as well as fingerprint evidence, can be difficult because each piece of evidence can have a differential evidentiary value, the method of quantifying the observed evidence in this study was in line with South Korean police practice. This coding method can be simply defined as counting the number of evidence files, implying that the evidentiary or quality of the evidence was not reflected in the calculation.

#### 2.3.3 Investigation (referral) decision

The South Korean criminal justice system is an inquisitorial system in which police conduct thorough investigations and gather evidence. This evidence is then reviewed by the prosecutor, who decides whether to refer to a case for prosecution. After investigating a case, the police can make one of the following five investigative decisions: a) the decision to refer a case for prosecution (Referred for Prosecution), b) the decision to forward a case to a summary trial (Referred for Prosecution), c) the decision not to refer a case for prosecution because of the lack of sufficient or unconstitutionality of a crime (Not Referred), d) the decision to close a case without an investigation (Not Referred), and e) the decision for an unsolved case (when a suspect is not identified; Not Referred). Finally, based on the team's five decisions, the 172 criminal cases were categorized into the following dichotomy for further analysis: i) Referred for Prosecution and ii) Not Referred.

## 3 Results

### 3.1 Descriptive statistics

#### 3.1.1 Crime type

**Fig. 1:**
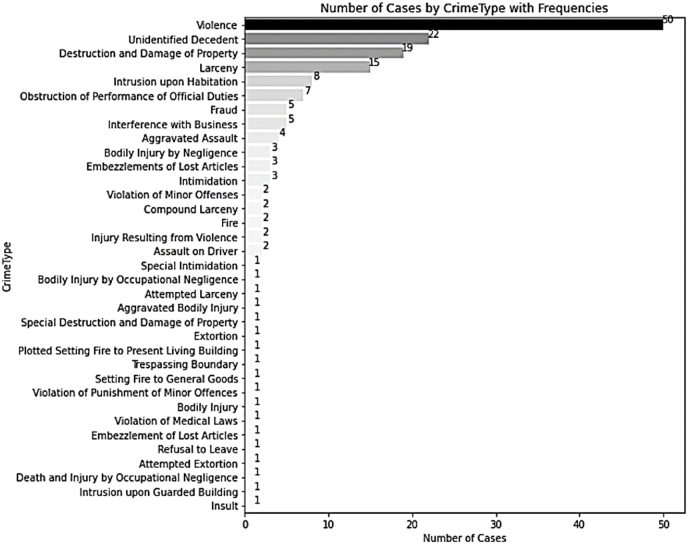
Distribution of Analyzed 172 Cases by Crime Type *Note. N* = 172 cases. The English name of the crime types were derived from the translated version of the South Korean Criminal Act published by the Korean Law Information Center (https://www.law.go.kr/). The crime types were arranged in descending order from the most observed crime type, violence.

The dataset used for the analyses included 172 reported incidents distributed across 35 crime classifications (see Fig. 1). The range of crime types included in the dataset is extensive, illustrating the varied nature of volume crimes handled by the investigation team. The most frequently occurring crime type within this dataset was ‘violence,’ which accounted for a substantial proportion of the dataset with 50 instances reported. This type of crime accounts for 29.1 % of the total number of crimes. Violence was followed by ‘unidentified decedent^3^’ (22 cases), ‘destruction and damage of property’ (19 cases), and ‘larceny’ (15 cases).

#### 3.1.2 Evidence collection by type

Table 2 shows a higher prevalence of the team's collection of testimonial evidence, such as victim statements (*M* = 1.15, *SD* = 0.56), suspect statements (*M* = 0.83, *SD* = 0.75), and witness testimonies (*M* = 0.76, *SD* = 0.96). In contrast, forensic evidence, such as DNA (*M* = 0.01, *SD* = 0.08), fingerprints (*M* = 0.01, *SD* = 0.08), and chemicals (*M* = 0.01, *SD* = 0.11) were the least collected types. Among the physical evidence types, video recording was the most common (*M* = 0.58, *SD* = 0.71). It was also found that the investigation team collected an average of four pieces of evidence per case (*M* = 3.95, *SD* = 2.26).

**Table 2:** Descriptive statistics of evidence collected in 172 volume crime cases.

Type	*M* (*SD*)	*Mdn*	Min	Max	CI (95 %)
Physical					
Video Recording	0.58 (0.71)	0.00	0.00	3.00	[0.47, 0.68]
Voice Recording	0.03 (0.21)	0.00	0.00	2.00	[0.00, 0.07]
Photo	0.44 (0.66)	0.00	0.00	3.00	[0.34, 0.54]
Document	0.35 (0.76)	0.00	0.00	5.00	[0.23, 0.46]
Digital Trace^a^	0.08 (0.31)	0.00	0.00	2.00	[0.03, 0.13]
DNA^a^	0.01 (0.08)	0.00	0.00	1.00	[-0.01, 0.02]
Fingerprint^a^	0.01 (0.08)	0.00	0.00	1.00	[-0.01, 0.02]
Chemicals^a^	0.01 (0.11)	0.00	0.00	1.00	[0.00, 0.03]
***Testimonial***					
Witness Testimony	0.76 (0.96)	0.00	0.00	4.00	[0.61, 0.90]
Victim Statement (*n* = 135)^b^	1.15 (0.56)	1.00	0.00	4.00	[1.05, 1.26]
Suspect Statement (*n* = 129)^b^	0.83 (0.75)	1.00	0.00	5.00	[0.71, 0.94]
Total Evidence	3.95 (2.26)	3.50	1.00	15.00	[3.61, 4.29]

*Note. N* = 172 cases.

a Denotes forensic evidence.

b These types of evidence were collected less due to the contingencies of the team's charge decision or the type of the crimes.

#### 3.1.3 Crime scene processing and forensic evidence collection

Descriptive statistics indicate that the CSI team was called in to process the crime scenes in only 30 of the 172 cases, including 22 unidentified death cases; only one piece of DNA and one fingerprint were found by the CSI team in one of the 30 cases. In addition, one piece of chemical evidence was collected from two cases of the 30 cases.

### 3.2 Evidence collection before and after interrogation

Descriptive statistics show that 66.7 % of the evidence was collected before the interrogation and 33.3 % was collected after the interrogation (see Table 3). A paired samples *t*-test was conducted to compare the amount of evidence before (*M* = 2.95, *SD* = 1.58) and after interrogation (*M* = 1.44, *SD* = 1.27) across 123 cases in which the team had interrogated the suspect. There was a significant difference in the amount of evidence collected before and after the interrogation (*t*[122] = 10.26, *p* < 0.001), indicating that a larger amount of evidence was typically obtained prior to the interrogation.

**Table 3:** Descriptive statistics of evidence before interrogation in 129 volume crime cases.

	Type	*M* (*SD*)	*Mdn*	Min	Max
Before Interrogation	***Physical***	1.37 (1.21)	1.00	0.00	6.00
	Videos	0.50 (0.64)	0.00	0.00	3.00
	***Testimonial***	1.57 (1.01)	1.00	0.00	5.00
	Eyewitness Testimony	0.53 (0.80)	0.00	0.00	3.00
	**Available Evidence (a)**	2.94 (1.57)	3.00	0.00	10.00
After Interrogation	**Additional Evidence (b)**	1.47 (1.26)	1.00	0.00	7.00
	Total Evidence (a + b)	4.40 (2.31)	4.00	1.00	15.00

*Note*. *n* = 129 cases. In 129 of 172 cases, the detectives conducted an interrogation.

### 3.3 Correlation between video recording and eyewitness testimony evidence

**Fig. 2:**
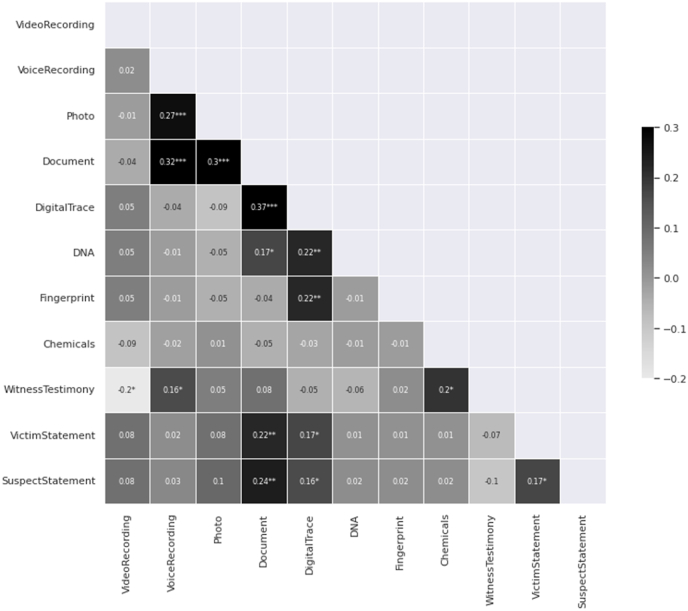
Correlation Matrix of the 11 Types of Evidence Collected *Note. N =*172 cases. This figure presents the correlation matrix of 11 evidence variables in the study. Each cell represents the correlation coefficient between a pair of variables, with the shading indicating the strength and direction of the correlation. Darker cells denote high positive correlations, while lighter cells signify negative correlations. *p < 0.05. **p < 0.01. ***p < 0.001.

The correlation analysis between the presence of video recording evidence and witness testimony in the cases revealed a slight negative relationship, *r*(170) = −0.196, *p* < 0.05 (see Fig. 2). This suggests that cases with video recording evidence are slightly less likely to contain witness testimonies. The effect size (Cohen's *d*) for the negative relationship between the presence of video recording evidence and witness testimony was −0.40. The 95 % confidence interval for this effect size ranged from −0.71 to −0.10, indicating a small to medium effect. This suggests that the true effect size could be as small as −0.10 or as large as −0.71 with 95 % confidence.

### 3.4 Evidence collection by investigation decision

Table 4 shows that 132 cases were identified as having a crime suspicion, excluding non-crime cases from these analyses (i.e., unidentified decedent; those cases that were identified as not being a crime). The investigation team made referral decisions for 132 cases (whether to be forwarded to the prosecution's office). The descriptive statistics show that the detective team referred 72 cases to the prosecution, whereas 60 cases were not referred. The quantity of evidence collected was compared based on these two referral decisions (see Table 4 for further details).

**Table 4:** Descriptive statistics of collected evidence by referral decision for prosecution.

Category	Type	Decision	*n*	*M*	*SD*
Physical	Video Recording	Referred	72	0.76	0.68
		Not Referred	60	0.30	0.46
	Voice Recording	Referred	72	0.04	0.20
		Not Referred	60	0.03	0.26
	Photo	Referred	72	0.43	0.62
		Not Referred	60	0.48	0.72
	Document	Referred	72	0.46	0.92
		Not Referred	60	0.32	0.65
	Digital Trace	Referred	72	0.13	0.41
		Not Referred	60	0.05	0.22
	DNA	Referred	72	0.01	0.12
		Not Referred	60	0.00	0.00
	Fingerprint	Referred	72	0.01	0.12
		Not Referred	60	0.00	0.00
	Chemicals	Referred	72	0.01	0.12
		Not Referred	60	0.02	0.13
Testimonial	Witness Testimony	Referred	72	0.76	1.03
		Not Referred	60	0.68	0.91
	Victim Statement	Referred	72	1.21	0.71
		Not Referred	60	0.90	0.51
	Suspect Statement	Referred	72	1.07	0.59
		Not Referred^a^	57	0.93	0.84
	Total Evidence	Referred	72	4.90	2.21
		Not Referred	60	3.67	2.11

*Note*. *n* = 132 case. All these cases were filed a charge because their suspicion of crime involvement was perceived to be substantiated by sufficient evidence by the team.

a Three suspects did not provide a written statement because they were in prison or their whereabout was unknown.

**Fig. 3:**
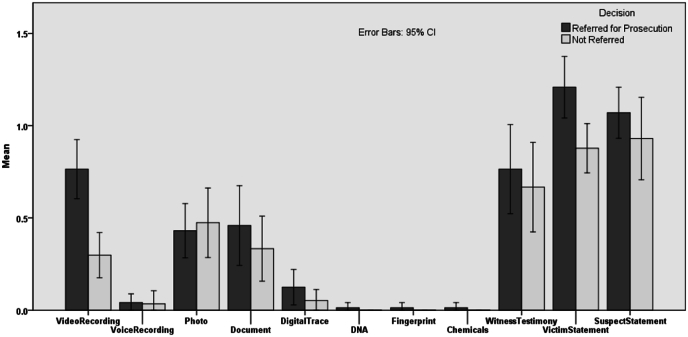
Mean Values of Evidence Collected by Investigation Decision *Note. N* = 172 cases. This bar graph shows the mean values for each type of evidence collected during investigations. The graph is divided into two sections: physical evidence types are aligned on the left side, while testimonial evidence types are displayed on the right side.

For the video recording variable, a *t*-test result indicated that there was a statistically significant difference in the collection of video recording evidence between the groups referred and not referred for prosecution, *t*(130) = 4.48, *p* < 0.001. This suggests that the investigation team collected more video evidence in the referred cases (vs. not-referred cases). An additional *t*-test revealed a statistically significant difference in victim statements between the two groups, *t*(130) = 2.81, *p* = 0.006, indicating that more victim statements were collected in the referred than in the not-referred cases. The other variables did not show any statistically significant differences based on the conventional alpha level of 0.05 (see Fig. 3).

### 3.5 Suspect's exonerating evidence

Suspects submitted exonerating evidence to the investigation team in 18 of 129 cases either before or after interrogation. In general, they tended to submit less than one piece of evidence (*M* = 0.32, *SD* = 0.84, *Mdn* = 0.0, Min = 0.0, Max = 4.0).

## 4 Discussion

The findings presented in this study shed new light on various aspects of different types of evidence collection and the subsequent decision-making processes within the investigative context. Researchers have suggested that evidence can be used in diverse ways. For example, many legal psychologists on the Strategic Use of Evidence (SUE) technique have found that disclosing evidence in strategic ways can aid interviewers in the context of lie detection and police interrogations [[34], [35], [36], [37]]. In addition, research on evidence judgements shows that legal professionals and laypeople tend to give more evidentiary weight to forensic (vs. testimonial) evidence [6,15,18]. Nevertheless, there is a notable gap in the academic literature concerning the collection of physical and testimonial evidence during criminal investigations. Field police investigators, due to their practical experience, often have a more comprehensive understanding of these processes compared to researchers. Therefore, this study aims to bridge this gap by providing researchers with deeper insights into the patterns of evidence collection, thereby informing and guiding future research in this area.

### 4.1 General patterns of evidence collection

The findings suggest that testimonial evidence such as victim statements, suspect statements, and witness testimonies emerged as the predominant form of evidence in the investigated cases where the offender was known. Forensic evidence, including DNA, fingerprints, and chemicals, was rarely collected. Nonetheless, video recording was the most prevalent type of physical evidence, suggesting its importance in contemporary investigative practice.

Volume crimes normally occur in public areas [38] and are not premeditated by perpetrators who do not care about whether they are observed by third party persons in the commission of a crime. I found that testimonial (vs. physical) evidence was comparatively easier to collect in volume crime cases. For example, in an interpersonal violence case, even patrol officers who were dispatched to the scene often witnessed the crime and provided a witness statement to the detective team. In addition, investigators can lessen their efforts to close a case by interviewing eyewitnesses at the scene because the information provided by eyewitnesses at a scene can lead to quicker and more efficient follow-up investigations, eventually resulting in arrests [29]. The data may have reflected the practicality of gathering on-scene information, even at the start of crime occurrence.

Detectives seek to find video recording evidence because they have more powerful evidentiary values than any other type of evidence [24,25]. A video capturing how a crime has occurred can help detectives make prompt decisions owing to its easy operational accessibility. For instance, in some cases, it can be more convenient for detectives to collect video recordings instead of finding an eyewitness. In most cases, finding an eyewitness requires a tremendous workforce; it may take the same amount of energy to identify who witnessed the crime as to find a perpetrator. In comparison, if investigators see a public CCTV camera installed around a crime scene, they can easily access the video file by visiting the government office in charge. For private CCTVs, detectives should ask for permission from the CCTV owners.

Citizens may hesitate to cooperate with police investigators because providing cooperation with the police is time-consuming or they fear potential retaliation [[29], [39], [40]]. In a metropolitan city, citizens live busy lives, and they need to spend one or 2 h making their statements written on an investigative dossier. In addition, they do not want to be involved in a crime for someone they do not know, and it may not matter to them how innocent they believe the victim is. Therefore, it is presumable that, because of this utility, detectives prefer to find CCTV cameras first and foremost, turning to other evidence when any CCTV is not available.

However, it is noteworthy that criminal investigators can encounter numerous obstacles before, during, and after collecting CCTV footage [[41], [42]]. For instance, investigators often face difficulties in accessing footage in camera control rooms, transferring video files, dealing with limited storage capacity, establishing cooperation with various stakeholders, and addressing file compatibility and systematic errors. Analyzing lengthy videos poses another tremendous challenge. Notably, recognizing a suspect's face in video evidence can be problematic because of the varying quality of CCTV cameras [43], particularly when suspects deny their presence in footage. Considering the potential inaccuracies of CCTV video evidence, investigators should be cautious of its quality and avoid relying solely on video evidence to solve their cases.

### 4.2 Lack of evidence

Some results in this study show that the detective team was able to collect two pieces of evidence per case on average, except for victim and suspect statements: potentially video recording and witness testimony. This implies that in practice, investigators have less evidence than we can expect. This is primarily because finding evidence in real-life scenarios is more difficult than finding evidence in CSI dramas. Collecting physical evidence at a crime scene is more difficult than testimonial evidence. This trend may result from the fact that patrol officers hesitate to protect or barricade scenes with low certainty of suspicion, because volume crimes are less serious in nature and do not have tangible on-scene traces or evidence [44]. Imagine that in a busy morning, police officers of a metropolitan city such as Seoul cannot stop commuters from entering a crime scene of minor crime in a congested subway station without obvious doubt that the reported incident is an actual crime. Likewise, it is highly difficult for uniform officers to make accurate judgements of whether the crime meets its criminality criteria or whether a suspect has really committed the crime.

Police detectives are typically overloaded with multiple cases and under time pressure, which can influence their cognitive abilities [[45], [46], [47]]. Thus, detectives can strategically choose to invest more time in solving other cases instead of collecting additional evidence for a volume crime. Also, proving the elements of volume crime (corpus delicti) is comparatively easier than other types, which can relieve detectives' psychological pressure to find more evidence that incriminates or exonerates a suspect's suspicion in the initial phase of an investigation. The current study's data reveal that a larger proportion of evidence was gathered before the interrogation, which could be related to these types of motivational or environmental factors affecting the behavior to collect evidence. However, the specific factors that contribute to this trend are not yet clear. Future research should explore whether this finding is limited to the current study and what can explain this difference if this trend is observed in additional samples.

### 4.3 Potential factors affecting forensic evidence collection

The limited instances of DNA, fingerprints, and chemical evidence collection indicate potential challenges in forensic evidence acquisition, which aligns with previous studies [44,48]. There are several potential explanations for this finding.

Volume crimes are the most prevalent types of offenses encountered by police on a daily basis, and their severity is relatively low compared to serious crimes such as murder, robbery, and rape. Consequently, in practice, volume crimes are not prioritized in forensic examinations. In South Korea, most volume crime cases are assigned to detectives with pre-existing information about the suspect's identity. This can lead to reduced motivation to involve CSI agents, as the collection of physical evidence may not impact clearance rates [3]. In addition, the traditional intelligence-led policing environment may not be ready for being integrated with the science-led investigation [19].

Considering the lack of human resources in police forces, CSI agents are not able to process crime scenes as often as they are requested. Kim [49] reported that there are 1417 CSI agents working under the Bureau of Forensic Investigation, in KNPA. Of these, only 226 agents are responsible for covering the entirety of Seoul, a densely populated capital city. In South Korea, CSI teams handle both major and volume crimes, ensuring comprehensive coverage of all types of criminal investigations. Prioritization of cases is typically based on the severity and urgency of the crime, with major crimes such as homicides, armed robbery, and rape receiving immediate attention, while volume crimes are addressed based on available resources and detectives’ request. Processing a crime scene is time-consuming and laborious; thus, investigation departments may be under pressure to allocate CSI teams to serious crimes more often.

In turn, the detectives become concerned about potential delays in their investigation. Detectives are required to make a final decision regarding a case within a certain period of time [44]. As soon as a case is assigned to them, the time ticks; victims and reporters want their case finalized and can pressure detectives to clear it as soon as possible. Forensic evidence should be sent to a forensic laboratory, and it takes a while to receive its analysis report [10]. Given this pressure, detectives may be less motivated to spend time collecting evidence [44]. The findings of the current study support this argument that the detective team summoned the CSI team only for 30 out of the 172 cases. Since the CSI team was present at each of the requested crime scenes, it can be reasonably inferred that more than 30 crime scenes could have been processed. Among these cases, the majority were unidentified decedent cases where detectives must call CSI teams to process the deceased's bodies and scenes to determine the potential causality of a crime (i.e., homicide), and the CSI analysis report should be attached to the investigative documents for a referral decision. Considering the size of the population in Seoul and the high demand for forensic services, it is evident that the current number of CSI agents is insufficient. To enhance the effectiveness and efficiency of forensic investigations, it is imperative for the government to recruit additional CSI agents to adequately meet the needs of such a large urban area.

Crime scene processing is known to be costly, which might have affected the data in the current study. The cost of processing a case involving DNA evidence, from the delivery of the evidence to the local forensic laboratory to the testing of a suspect's confirmation sample, ranges approximately from $800 to $2400 [50]. In theory, at a crime scene, a CSI agent can gather numerous evidence samples and submit them for DNA or other types of forensic analysis. Considering the substantial increase in the amount of forensic evidence analyzed by the Korean National Forensic Services [28], the budget that the South Korean government invests every year in processing forensic evidence may be increasing accordingly. Although assessing the cost-effectiveness of forensic evidence to maintain social security (i.e., arrest, conviction, and clearance rates) presents challenges due to the scarcity of data [51], it is plausible to infer that the extensive collection of evidence imposes a financial burden on the government and KNPA. Consequently, for economic reasons, the practice of gathering substantial amounts of evidence may be discouraged, and this may affect investigators' collection of evidence in volume crime cases.

In addition, detectives and CSI agents may have some communication barriers. In South Korea, CSI agents and detectives work in separate departments, meaning they are under different bureaucratic hierarchies: Detectives are under the Bureau of Criminal Investigation, and CSI agents are under the Bureau of Forensic Investigation. Maintaining the separate system is the crucial part in the criminal justice system. However, this structural difference may create operational inconvenience in terms of the communication channels such as having different chains of command, working regulations, and rules to follow. Therefore, conducting further research to minimize the impact of organizational structures on collaboration and information exchange could offer valuable insights for optimizing investigative processes.

Preliminary investigations and physical evidence collection at crime scenes are primarily the responsibility of patrol officers, the first responders. Crime scene specialists also play a role in evidence collection, and there is a suggestion that their knowledge and skills may need to be utilized more effectively because police officers and forensic investigators are known to have variations in their reasoning, knowledge, and training in terms of evidence collection and forensic expertise [52]. Additionally, managing crime scenes can be complex due to the involvement of multidisciplinary and multi-organizational personnel, who often have divergent work practices and views on their roles during investigations and court trials [19].

In this study, four detectives in the team, excluding the author, had no opportunity to receive training or education about forensic science before the study began. This lack of forensic knowledge might have affected the trend of evidence collection during their investigations. Detectives in criminal investigation departments in South Korea are the ones who decide whether scenes are processed by CSI agents, and their decisions at the initial stages of an investigation can be critical to the overall outcomes of justice [19]. Some studies in South Korea have reported noticeable variations in the training and education levels of forensic knowledge among CSI investigators, as well as between CSI investigators and other police officer groups in South Korea [[49], [56]]. This disparity in training and expertise among detectives, police officers, and CSI personnel represents a limitation that warrants attention. The effectiveness of evidence collection is likely influenced by the professional background and training of the involved personnel, suggesting a need for standardized training protocols and guidelines to ensure consistent and reliable forensic practices. Also, KNPA needs to review and establish clear guidelines for CSI attendance at crime scenes to help detectives who are often overloaded with investigations and under time pressure.

A considerable proportion may not be subjected to scientific analysis, despite the collection of physical evidence [6,11]. Evidence is mainly submitted for laboratory analysis by specialists, but the extent to which collected forensic evidence is sent and analyzed varies widely among jurisdictions [19], emphasizing the need for further exploration of the factors that influence these processes.

Lastly, the introduction of the President's Council of Advisors on Science and Technology (PCAST) findings into South Korean police practices, particularly in the crime scene investigation sector, needs to be taken into account, which can potentially enhance the scientific rigor and reliability of forensic methods used. PCAST's critical evaluation of existing forensic science research revealed substantial methodological weaknesses, leading to questions about the validity and reliability of forensic evidence in legal contexts [53]. Implementing PCAST's recommendations, such as the adoption of registered reports, would ensure that forensic methodologies undergo rigorous peer review before data collection, promoting transparency and reducing biases. This could improve the credibility of forensic practices within South Korean crime scene investigations, aligning them with international standards and enhancing their acceptance in judicial processes [[53], [54]].

## 5 Limitations and future research

Although this study provides valuable insights into the patterns of evidence collection in conjunction with decision-making processes within the investigative context, several limitations should be considered. As noted above, the findings were based on a specific dataset of 172 reported incidents within a particular context, limited to one volume crime investigation team in a densely populated region of Seoul Metropolitan City, South Korea. Generalizing these results to broader populations or diverse jurisdictions may be challenging. Future studies should replicate these findings across different cities and countries to establish the generalizability of the observed patterns. The dataset covers 35 crime classifications; however, certain types may be underrepresented or omitted. Exploring the nuances of evidence collection in specific crime categories, such as felony crime, sexual crime, cybercrime, or white-collar crime, could provide a more comprehensive understanding of investigative practices. The study did not extensively delve into contextual factors influencing evidence collection, such as socioeconomic conditions, cultural aspects, or legal frameworks. Future research should explore how these contextual factors shape patterns of evidence collection and investigative decision-making.

These limitations suggest avenues for future research to enhance our understanding of the dynamics involved in criminal investigation. Future research could examine additional contextual factors that affect evidence collection and legal decisions, thereby contributing to a more comprehensive understanding of the investigative processes and ensuring a fair and effective criminal justice system.

## 6 Conclusion

This study provides a valuable foundation for understanding the patterns and challenges of evidence collection in criminal investigations. While these findings contribute potentially to the existing literature, it is imperative to acknowledge the limitations outlined. Addressing these limitations through future research will not only enhance the robustness of the observed patterns but also contribute to the development of more effective investigative practices. The intricate interplay between evidence type, crime type, availability of evidence, and decision-making processes warrants continuous exploration to ensure the fairness and efficiency of criminal investigations.

## CRediT authorship contribution statement

**Minhwan Jang:** Writing – review & editing, Writing – original draft, Visualization, Validation, Supervision, Software, Resources, Project administration, Methodology, Investigation, Formal analysis, Data curation, Conceptualization.

## Declaration of generative AI and AI-assisted technologies in the writing process

During the preparation of this work, the author(s) used ChatGPT and Paperpal for grammar and spell checks. Julius was used only for statistical analyses and generating images of results. After using these tools/services, the author(s) reviewed and edited the content as needed and take(s) full responsibility for the content of the publication.

## Declaration of competing interest

I have no known conflict of interest to disclose.

## Footnotes

1 Police charge in this paper is a decision made by an investigator(s) before transferring a case to prosecution in relation to whether to conduct an initial investigation of a reported crime. The police charge is a legal term as ‘Ip-geon’ in Korean. When a detective believes that a reported incident is alleged to be or suspicious of a crime, the detective needs to file a charge against a suspect(s) to make further investigations.

2 The total number of crimes reported to the police in 2011 was 1,752,598, whereas in 2022, the number decreased to 1,482,433 KNPA, 2023a.

3 An Unidentified Decedent in South Korea refers to a deceased person whose identity or cause of death is unknown at the time of discovery or investigation. The unidentified decedent is a legal and practical term, which can typically be called as natural or accidental death or suicide. This case is typically handled by volume crime teams. This situation typically arises when a body is found, but no immediate information is available about whether the deceased died from criminal activity. Authorities such as police forces, prosecutors, or forensic experts work to determine an individual's identity and cause of death through various means, including fingerprint analysis, dental records, DNA testing, and witnesses to gather information. Once a person's identity is established, further investigation into the circumstances of their death can proceed, and efforts can be made to inform and involve their family or next of kin. When the death is found to have resulted from any criminal activity, the case is reassigned to a felony investigation team and is no longer considered as a volume crime.

## References

[1] Korean National Police Agency (2024). Korean national police agency organizational structure 2024. https://www.police.go.kr/eng/knpa/org/org01.jsp.

[2] Bradbury S.A., Feist A. (2005).

[3] rådet Brottsförebyggande (2013). Police officers' view of the investigation of volume crime: assignment “Investment in more police officers”. https://www.bra.se/bra-in-english/home/publications/archive/publications/2013-12-20-police-officers-view-of-the-investigation-of-volume-crime.html.

[4] Association of Chief Police Officers (2009). Wyboston: National Policing Improvement Agency.

[5] Houck M.M., Siegel J.A. (2009).

[6] Peterson J.L., Hickman M.J., Strom K.J., Johnson D.J. (2013). Effect of forensic evidence on criminal justice case processing. J. Forensic Sci..

[7] Wüllenweber S., Giles S.B. (2021). The effectiveness of forensic evidence in the investigation of volume crime scenes. Sci. Justice.

[8] Brown C., Ross A., Attewell R.G. (2014). Benchmarking forensic performance in Australia–volume crime. Forensic Sci. Pol. Manag.: Int. J..

[9] Braga A.A., Turchan B., Barao L. (2019). The influence of investigative resources on homicide clearances. J. Quant. Criminol..

[10] Bruenisholz E., Vandenberg N., Brown C., Wilson-Wilde L. (2019). Benchmarking forensic volume crime performance in Australia between 2011 and 2015. Forensic Sci. Int.: Synergy.

[11] Peterson J.L., Sommers I., Baskin D., Johnson D. (2010). https://www.ojp.gov/pdffiles1/nij/grants/231977.pdf.

[12] Jang M., Luke T.J., Granhag P.A., Vrij A. (2020). The impact of evidence type on police investigators' perceptions of suspect culpability and evidence reliability. Zeitschrift Fur Psychologie.

[13] Alderden M.A., Ullman S.E. (2012). Creating a more complete and current picture: examining police and prosecutor decision-making when processing sexual assault cases. Violence Against Women.

[14] O'Neal E.N., Tellis K., Spohn C. (2015). Prosecuting intimate partner sexual assault: legal and extra-legal factors that influence charging decisions. Violence Against Women.

[15] Appleby S.C., Kassin S.M. (2016). When self-report trumps science: effects of confessions, DNA, and prosecutorial theories on perceptions of guilt. Psychol. Publ. Pol. Law.

[16] Golding J.M., Stewart T.L., Yozwiak J.A., Djadali Y., Sanchez R.P. (2000). The impact of DNA evidence in a child sexual assault trial. Child. Maltreat..

[17] Lieberman J.D., Carrell C.A., Miethe T.D., Krauss D.A. (2008). Gold versus platinum: do jurors recognize the superiority and limitations of DNA evidence compared to other types of forensic evidence?. Psychol. Publ. Pol. Law.

[18] Pearson J.M., Law J.R., Skene J.A., Beskind D.H., Vidmar N., Ball D.A., Skene J.P. (2018). Modelling the effects of crime type and evidence on judgments about guilt. Nat. Human Behav..

[19] Julian R., Kelty S., Robertson J. (2012). “Get it right the first time”: critical issues at the crime scene. Curr. Issues Crim. Justice.

[20] Baskin D.R., Sommers I.G. (2010). The influence of forensic evidence on the case outcomes of homicide incidents. J. Crim. Justice.

[21] Baskin D., Sommers I. (2012). The influence of forensic evidence on the case outcomes of assault and robbery incidents. Crim. Justice Pol. Rev..

[22] Porter G. (2009). CCTV images as evidence. Aust. J. Forensic Sci..

[23] Brookman F., Jones H. (2021). Capturing killers: the construction of CCTV evidence during homicide investigations. Polic. Soc..

[24] Dowling C., Morgan A., Gannoni A., Jorna P. (2019). How do police use CCTV footage in criminal investigations?. Trends and Issues in Crime and Criminal Justice.

[25] Morgan A., Dowling C. (2019).

[26] Lee H.K. (2021). Interpretation and legislation methodology on the seizure of CCTV video information with consent. Journal of Korean Lawyer Association.

[27] Korean National Police Agency (2023). https://www.police.go.kr/user/bbs/href=/component/file/ND_fileDownload.do?q_fileSn=156695&q_fileId=eddcb193-3702-456c-8fc7-becb905b48f8.

[28] Korean National Forensic Service (2024). Yearly report on statistics on forensic analyses 2024. https://nfs.go.kr/site/nfs/stat/selectWebLogStatNfs.do.

[29] McEwen T., Regoeczi W. (2015). Forensic evidence in homicide investigations and prosecutions. J. Forensic Sci..

[30] Kebbell M.R., Milne R. (1998). Police officers' perceptions of eyewitness performance in forensic investigations. J. Soc. Psychol..

[31] Wells G.L., Memon A., Penrod S.D. (2006). Eyewitness evidence: improving its probative value. Psychol. Sci. Publ. Interest.

[32] Boyce M.A., Lindsay D.S., Brimacombe C.A.E. (2008). Investigating investigators: examining the impact of eyewitness identification evidence on student-investigators. Law Hum. Behav..

[33] Seoul Metropolitan Government (2024). City overview: population. https://english.seoul.go.kr/seoul-views/meaning-of-seoul/4-population/.

[34] Oleszkiewicz S., Watson S.R. (2020). A meta-analytic review of the timing for disclosing evidence when interviewing suspects. Appl. Cognit. Psychol..

[35] Granhag P.A., Hartwig M., Granhag P.A., Vrij A., Verschuere B. (2015). Deception Detection: Current Challenges and New Approaches.

[36] Luke T.J., Dawson E., Hartwig M., Granhag P.A. (2014). How awareness of possible evidence induces forthcoming counter-interrogation strategies. Appl. Cognit. Psychol..

[37] Moston S., Engelberg T. (2011). The effects of evidence on the outcome of interviews with criminal suspects. Police Pract. Res..

[38] Korean National Police Agency (2023). Yearly Report on Statistics on Crime Trends in Places 2022.

[39] Lino D., Roazzi A., Viale R. (2024). Investigative decision-making: a qualitative analysis of homicide detectives' practices. Homicide Stud..

[40] Regoeczi W.C., Jarvis J.P. (2013). Beyond the social production of homicide rates: extending social disorganization theory to explain homicide case outcomes. Justice Q. JQ.

[41] Keval H., Sasse M.A. (2010). “Not the usual suspects”: a study of factors reducing the effectiveness of CCTV. Secur. J..

[42] Mangku D.G.S., Yuliartini N.P.R., Dewi K.R., Arta K.S. (2022). Disclosure of crime cases through CCTV: how does technology help police performance?. AIP Conf. Proc..

[43] Macarulla Rodriguez A., Geradts Z., Worring M., Unzueta L. (2024). Improved likelihood ratios for face recognition in surveillance video by multimodal feature pairing. Forensic Sci. Int.: Synergy.

[44] Tilley N., Robinson A., Burrows J., Newburn T., Williamson T., Wright A. (2007). The Handbook of Criminal Investigation.

[45] Akinola M., Mendes W.B. (2012). Stress-induced cortisol facilitates threat-related decision making among police officers. Behav. Neurosci..

[46] Almazrouei M.A., Dror I.E., Morgan R.M. (2020). Organizational and human factors affecting forensic decision-making: workplace stress and feedback. J. Forensic Sci..

[47] Ask K., Granhag P.A. (2005). Motivational sources of confirmation bias in criminal investigations: the need for cognitive closure. J. Investigative Psychol. Offender Profiling.

[48] Horvath F., Meesig R.T. (1996). The criminal investigation process and the role of forensic evidence: a review of empirical findings. J. Forensic Sci..

[49] Kim J. (2018). A study on the promotion of crime scene investigator - focused on the selection and training process -. Korean Journal of Public Safety and Criminal Justice.

[50] Roman J.K., Reid S.E., Chalfin A.J., Knight C.R. (2009). The DNA field experiment: a randomized trial of the cost-effectiveness of using DNA to solve property crimes. J. Exp. Criminol..

[51] Ludwig A. (2016). E ‘value’ ating Forensic Science. Forensic Sci. Pol. Manag.: Int. J..

[52] Ericson R. v, Shearing C.D., Böhme G., Stehr N. (1986). The Knowledge Society: the Growing Impact of Scientific Knowledge on Social Relations.

[53] Houck M.M., Chin J., Swofford H., Gibb C. (2022). Registered reports in forensic science. R. Soc. Open Sci..

[54] Ballantyne K.N., Summersby S., Pearson J.R., Nicol K., Pirie E., Quinn C., Kogios R. (2024). A transparent approach: openness in forensic science reporting. Forensic Sci. Int.: Synergy.

[55] Alderden M., Cross T.P., Vlajnic M., Siller L. (2021). Prosecutors' perspectives on biological evidence and injury evidence in sexual assault cases. J. Interpers Violence.

[56] Jeon Y., Kim S., Seo W., Kim Y. (2018). A study on the improvement of scientific investigation division through the citizen participation organizational diagnosis. Korean Police Studies Review.

[58] Oatley G., Chapman B., Speers J. (2020). Forensic intelligence and the analytical process. WIREs: Data Min. Knowl. Discov..

[59] e-Nara Index (2023, May 18). Public CCTV Installations and Management https://www.index.go.kr/unity/potal/main/EachDtlPageDetail.do?idx_cd=2855. (Accessed 13 April 2024).

